# Supplementary material for: Molecular Characterization of Two Totiviruses from the Commensal Yeast Geotrichum candidum
Source: Viruses. 2023 Oct 25;15(11):2150. doi: 10.3390/v15112150 (PMC10674808; doi:10.3390/v15112150)
Supplement: Supplementary file 1 [file viruses-15-02150-s001.zip › viruses-2636151-supplementary.pdf]

Table S1. Primers used for the RT-PCR amplifications of terminal sequences of the two dsRNAs (GcTV2-Gc6 and GcTV4-Gc6). Each primer was used in combination with primer T4LC.

DsRNA (virus)	Primer name	Primer sequence
dsRNA1 (GcTV2-Gc6)	GcTV2-3F	CTACATCATGTTGTAGGCGG
	GcTV2-5R	ATGTCAAGCTTAACATGACC
dsRNA2 (GcTV4-Gc6)	GcTV4-3F	CAACAGCCACACAACTGG
	GcTV4-5R	ACGTTGACTCTGTATCCC

Table S2. Results of Blastx search (accessed in October 2023) for dsRNA1 (Geotrichum candidum totivirus 2 isolate Gc6) sequence (top 50 hits are shown).

Virus	Query Cover	E value	Percent Identity	Accession number
Totiviridae sp.	44%	0.0	97.50%	WAK80738.1
Totiviridae sp.	44%	0.0	97.35%	WAK80740.1
Geotrichum candidum totivirus 2	44%	0.0	95.29%	UUT40376.1
Totiviridae sp.	46%	0.0	94.15%	WAK80739.1
Totiviridae sp.	46%	0.0	94.15%	WAK80737.1
Geotrichum candidum totivirus 2	52%	0.0	91.66%	UUT40377.1
Saccharomyces cerevisiae virus L-A	47%	0.0	57.38%	NP_620495.1
Saccharomyces cerevisiae virus L-A	96%	0.0	54.60%	QRN76664.1
Saccharomyces kudriavzevii virus L-A1	96%	0.0	54.60%	YP_009328931.1
Saccharomyces cerevisiae virus L-A	96%	0.0	54.36%	QRN76660.1
Saccharomyces cerevisiae virus L-A	96%	0.0	54.36%	QRN76662.1
Saccharomyces cerevisiae virus L-A-2	96%	0.0	54.36%	AHF27234.1
Saccharomyces cerevisiae virus L-A	96%	0.0	54.24%	QRN76658.1
Saccharomyces cerevisiae virus L-A	96%	0.0	54.24%	NP_620493.1
Saccharomyces cerevisiae virus L-A	96%	0.0	54.24%	Q87022.2
Saccharomyces paradoxus virus L-A-21	96%	0.0	54.12%	ATL63180.1
Saccharomyces paradoxus virus L-A-74	96%	0.0	54.12%	ATL63184.1
Saccharomyces cerevisiae virus L-A-lus	96%	0.0	54.12%	AFQ55381.1
Saccharomyces cerevisiae virus L-A-28	96%	0.0	53.88%	AMV49327.1

Virus	Query Cover	E value	Percent Identity	Accession number
Saccharomyces paradoxus virus L-A-62	96%	0.0	53.88%	ATL63192.1
Saccharomyces paradoxus virus L-A-1143	96%	0.0	53.88%	ATL63190.1
Saccharomyces kudriavzevii virus L-A-1082	96%	0.0	53.87%	ATL63196.1
Saccharomyces paradoxus virus L-A-4650	97%	0.0	53.79%	ATL63186.1
Saccharomyces uvarum virus L-A-10560	97%	0.0	53.67%	ATL63194.1
Saccharomyces cerevisiae virus L-A	54%	0.0	53.65%	AAA50321.1
Saccharomyces paradoxus virus L-A-1939	97%	0.0	53.55%	ATL63188.1
Saccharomyces paradoxus virus L-A-66	96%	0.0	53.17%	AYN80723.1
Saccharomyces paradoxus virus L-A-45	97%	0.0	52.84%	ATL63182.1
Pichia membranifaciens virus L-A	96%	0.0	52.59%	UNY77798.1
Torulaspora delbrueckii virus LA	96%	0.0	52.30%	QRN76668.1
Tuber aestivum virus 1	51%	0.0	52.20%	YP_009507833.1
Totiviridae sp.	96%	0.0	51.69%	UHS72452.1
Ambrosiozyma totivirus A	45%	0.0	50.22%	QED43028.1
Poaceae Liege totivirus 6	45%	0.0	44.15%	UVG55931.1
Saccharomyces paradoxus virus L-A-4650	42%	0.0	44.07%	ATL63185.1
Saccharomyces paradoxus virus L-A-1143	42%	0.0	44.07%	ATL63189.1
Saccharomyces paradoxus virus L-A-62	42%	0.0	44.07%	ATL63191.1
Saccharomyces paradoxus virus L-A-1939	42%	0.0	44.07%	ATL63187.1
Saccharomyces uvarum virus L-A-10560	42%	0.0	44.07%	ATL63193.1

Virus	Query Cover	E value	Percent Identity	Accession number
Saccharomyces paradoxus virus L-A-74	42%	0.0	43.92%	ATL63183.1
Hubei toti-like virus 2	45%	0.0	43.78%	YP_009336496.1
Saccharomyces kudriavzevii virus L-A1	42%	0.0	43.77%	YP_009328932.1
Saccharomyces cerevisiae virus L-A-28	42%	0.0	43.62%	AMV49326.1
Saccharomyces paradoxus virus L-A-66	42%	0.0	43.62%	AYN80722.1
Maize associated totivirus	50%	0.0	43.52%	QJD25939.1
Saccharomyces paradoxus virus L-A-21	42%	0.0	43.47%	ATL63179.1
Saccharomyces cerevisiae virus L-A	42%	0.0	43.47%	QRN76663.1
Saccharomyces cerevisiae virus L-A-lus	42%	0.0	43.47%	AFQ55380.1
Saccharomyces cerevisiae virus L-A	42%	0.0	43.47%	QRN76661.1
Saccharomyces cerevisiae virus L-A	42%	0.0	43.47%	QRN76659.1

Table S3. Results of Blastx search (accessed in October 2023) for dsRNA2 (Geotrichum candidum totivirus 4 isolate Gc6) sequence (top 50 hits are shown).

Virus	Query Cover	E value	Percent Identity	Accession number
Totiviridae sp.	46%	0.0	91.69%	WAK77686.1
Totiviridae sp.	46%	0.0	91.69%	WAK77295.1
Totiviridae sp.	52%	0.0	89.24%	WAK77685.1
Totiviridae sp.	52%	0.0	88.86%	WAK77294.1
Tuatara cloaca-assoiciated totivirus-3	18%	8e-123	66.79%	UUV42637.1
Totiviridae sp.	48%	0.0	54.47%	WAK77495.1
Xian Totiv tick virus 1	49%	0.0	54.41%	UYL95672.1
Totiviridae sp.	35%	0.0	53.93%	WAK77570.1
Totiviridae sp.	39%	0.0	52.02%	WAK77449.1
Malassezia restricta associated totivirus 1	45%	0.0	49.86%	CAI9858976.1

Virus	Query Cover	E value	Percent Identity	Accession number
Hanko totivirus 2	23%	8e-116	49.17%	UUV42486.1
Erysiphe necator associated totivirus 7	52%	0.0	48.05%	QJW70337.1
Erysiphe necator associated totivirus 6	52%	0.0	47.99%	QJW70335.1
Erysiphe necator associated totivirus 8	46%	0.0	47.05%	QJW70339.1
Delisea pulchra totivirus IndA	31%	7e-126	45.72%	AMB17475.1
Totiviridae sp.	99%	0.0	45.68%	UHS72518.1
Totivirus sp.	28%	5e-116	45.64%	QOQ37362.1
Malassezia globosa associated totivirus 1	50%	0.0	45.44%	CAI9858973.1
Scheffersomyces segobiensis virus L	99%	0.0	45.33%	YP_009507829.1
Scheffersomyces segobiensis virus L	56%	0.0	45.33%	YP_009507831.1
Malassezia sympodialis mycovirus	49%	0.0	45.20%	QNJ34609.1
Malassezia sympodialis mycovirus	92%	0.0	45.20%	QNJ34610.1
Dali Totiv tick virus 1	49%	0.0	45.14%	UYL95680.1
Erysiphe necator associated totivirus 8	52%	0.0	45.08%	WAK77400.1
Totiviridae sp.	24%	7e-100	44.65%	UHS72441.1
Totiviridae sp.	24%	5e-96	44.53%	UHS72410.1
Totiviridae sp.	49%	0.0	44.21%	WAK77568.1
Malassezia restricta virus MrV40L	55%	0.0	43.74%	QJA42331.1
Malassezia restricta virus MrV40L	98%	0.0	43.74%	QJA42332.1
Totiviridae sp.	51%	0.0	43.73%	WAK77406.1
Totiviridae sp.	51%	0.0	43.73%	WAK77396.1
Totiviridae sp.	44%	2e-175	43.67%	WAK77496.1
Xian Totiv tick virus 1	44%	3e-175	43.67%	UYL95673.1
Erysiphales associated mitovirus 1	52%	0.0	42.21%	QIP68035.1
Totiviridae sp.	43%	4e-170	42.21%	UHS72517.1
Scheffersomyces segobiensis virus L	43%	7e-170	41.86%	YP_009507830.1
Erysiphales associated totivirus 13	52%	0.0	41.76%	QIP68067.1

Virus	Query Cover	E value	Percent Identity	Accession number
Poaceae Liege totivirus 8	35%	9e-124	41.58%	UVG55935.1
Helianthus annuus leaf-associated totivirus 1	31%	4e-103	40.94%	UMQ74219.1
Dali Totiv tick virus 1	43%	2e-156	40.12%	UYL95681.1
Totiviridae sp.	43%	3e-154	40.06%	WAK77397.1
Totiviridae sp.	35%	4e-117	40.00%	WAK77555.1
Erysiphe necator associated totivirus 8	44%	1e-152	39.44%	WAK77401.1
Totiviridae sp.	44%	3e-150	39.44%	WAK77567.1
Phakopsora totivirus E	31%	3e-95	39.18%	QED42935.1
Erysiphales associated totivirus 1	52%	0.0	39.10%	QIP68044.1
Erysiphe necator associated totivirus 8	44%	9e-148	38.85%	QJW70340.1
Malassezia restricta virus MrV40L	43%	1e-150	38.60%	QJA42330.1
Erysiphe necator associated totivirus 7	42%	5e-143	38.53%	QJW70338.1
Malassezia sympodialis mycovirus	43%	1e-148	38.20%	QNJ34608.1

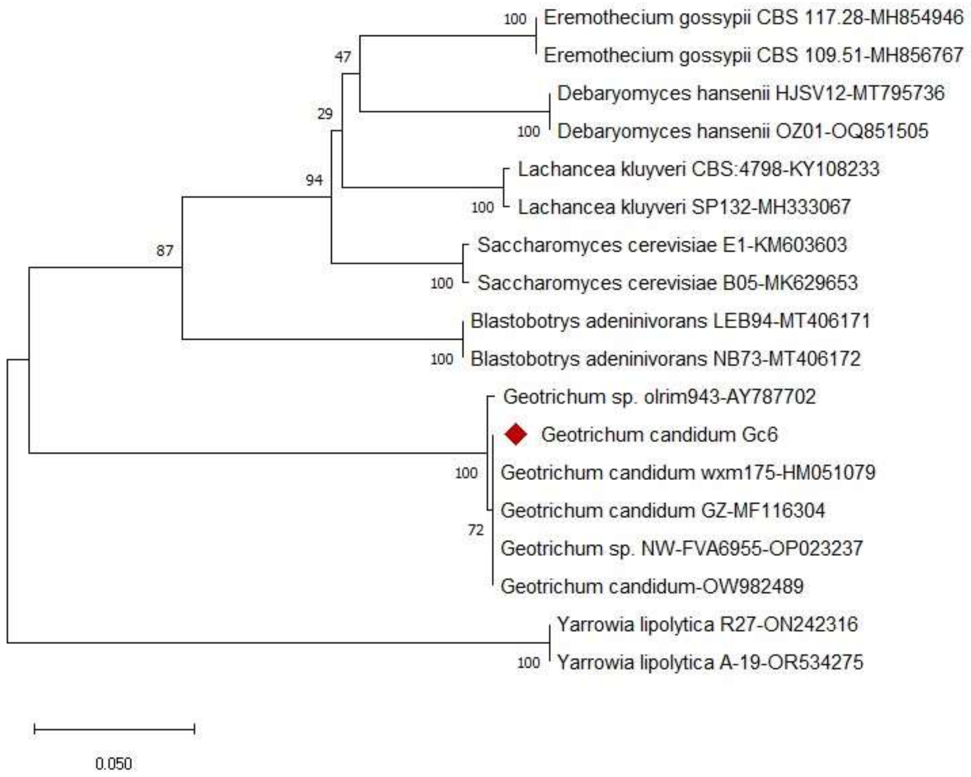

Figure S1. Neighbor-joining phylogenetic tree constructed based on partial ITS-5.8s rRNA gene sequence alignment of isolate Gc6 and other members of Saccharomycotina.
